# Bioconversion of Plastic Waste Based on Mass Full Carbon Backbone Polymeric Materials to Value-Added Polyhydroxyalkanoates (PHAs)

**DOI:** 10.3390/bioengineering9090432

**Published:** 2022-09-01

**Authors:** Brian Johnston, Grazyna Adamus, Anabel Itohowo Ekere, Marek Kowalczuk, Fideline Tchuenbou-Magaia, Iza Radecka

**Affiliations:** 1 Science in Industry Research Centre (SIRC), SciTech Innovation Hub, Wolverhampton Science Park, Glaisher Drive, Wolverhampton WV10 9RU, UK; 2 School of Science, Faculty of Science and Engineering, University of Wolverhampton, Wolverhampton WV1 1LY, UK; 3 Centre of Polymer and Carbon Materials, Polish Academy of Sciences, 41-800 Zabrze, Poland; 4 School of Engineering, Computing and Mathematical Sciences, Faculty of Science and Engineering, University of Wolverhampton, Wolverhampton WV1 1LY, UK

**Keywords:** polyethylene (PE), polypropylene (PP), polystyrene (PS), polyhydroxyalkanoate (PHA), poly(ethylene terephthalate) (PET), circular economy, *Cupriavidus necator*, bioplastics, bio-upcycling, Tetra Pak^®^

## Abstract

This review article will discuss the ways in which various polymeric materials, such as polyethylene (PE), polypropylene (PP), polystyrene (PS), and poly(ethylene terephthalate) (PET) can potentially be used to produce bioplastics, such as polyhydroxyalkanoates (PHAs) through microbial cultivation. We will present up-to-date information regarding notable microbial strains that are actively used in the biodegradation of polyolefins. We will also review some of the metabolic pathways involved in the process of plastic depolymerization and discuss challenges relevant to the valorization of plastic waste. The aim of this review is also to showcase the importance of methods, including oxidative degradation and microbial-based processes, that are currently being used in the fields of microbiology and biotechnology to limit the environmental burden of waste plastics. It is our hope that this article will contribute to the concept of bio-upcycling plastic waste to value-added products via microbial routes for a more sustainable future.

## 1. Introduction

**Figure 1:**
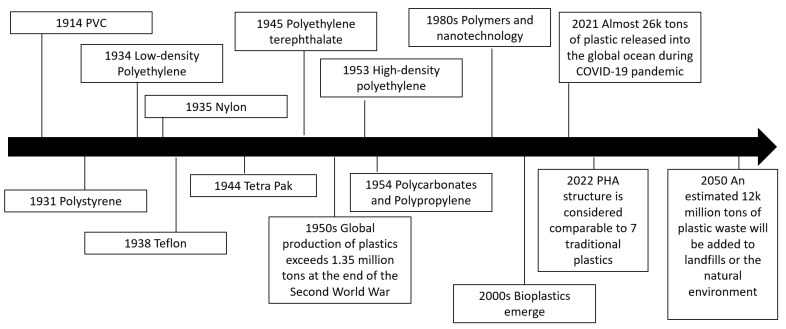
Milestones over the last century summarizing how bioplastics fit into the plastics industry.

The synthesis of polyhydroxyalkanoates (PHAs), a bioplastic that can be used to replace traditional (petrol-based) plastics, is an important focus in today’s politically and environmentally conscious society. PHAs are part of a group of organic polymers containing 3-, 4-, 5-, and 6-hydroxyalkanoic acids that are biocompatible, 100% biodegradable, and nontoxic to the environment [1,2]. PHAs can be considered a greener alternative to synthetic plastic compounds, and they can be produced by plants and various strains of bacteria (as documented later). In addition, these bioplastics can be generated through microbial fermentation using waste materials which could offer more sustainability in a closed-cycle system of carbon materials [2]. Some of the waste materials that have been used to make PHAs have included used synthetic plastics, such as PE, PS, and PP [2,3,4]. From 2022, by changing the co-monomer type and distribution in PHAs, the properties can be considered to be comparable with seven of the most profitable crude-oil-based plastics, which is estimated to be 230 million tons of plastic per year [5]. There have also been global policies put in place and capacity expansions for the next 5 years for over 1.4 million tons, so there is a lot of encouragement for the industry to adopt biomaterials [5,6]. It is also predicted that 12,000 million tons of plastic waste will be added to landfills or the natural environment by 2050 [7]. Figure 1 shows a timeline of milestones related to bioplastic development over the last hundred years.

Research projects around the world have focused on using microbes to break down some the most persistent types of plastics found in the environment. Currently, the most common way plastics are disposed of is by incineration, mechanical and chemical recycling, and the relatively cheap method of landfill sites [8]. However, all of these methods have their disadvantages; landfill occupies too much land space, and incineration creates secondary pollutants, such as dioxins and carbon monoxide. Even though mechanical recycling has become the main technique for refuge plastic, the chemical properties are usually compromised via processing, which results in reduced commercial value [9].

**Figure 2:**
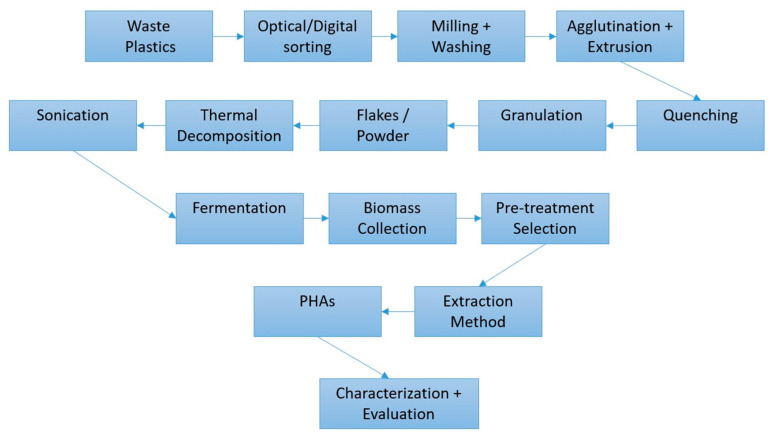
Potential operation stages for waste plastic material for the bio-generation of PHA bioplastics, adapted from [3]. The ‘Pre-treatment Selection’ of ‘Biomass’ process could include methods such as ionic liquid soaking, sonication, glass sphere mixing, and blade/pressure homogenization. Every procedure such as this in today’s economy should end with an evaluation step to ascertain any shortcomings and potential investigation avenues.

Chemical recycling is known to be able to recover monomers from plastic waste, but its success depends heavily on the efficiency of catalysts [9]. With up to 79% of waste plastic being discarded into landfills (or the surrounding environment), there is a huge requirement for novel recycling methodologies, and bioconversion is one possible answer [4,10,11]. The transfer of current feedstocks could be smoother if the true economical value (including the carbon footprint of products and practical benefits) were considered in detail. The efficiency of biotechnological methods can also be further improved using metabolic engineering, which could help achieve the aims of the internationally agreed Paris Agreement, a treaty on climate change [10]. Moreover, the recently estimated impact of the COVID-19 pandemic on plastic discharge indicates that around 8.4 million tons of pandemic-associated plastic waste was generated from 193 countries, as of 23 August 2021, and over 25.9 thousand tons were released into the global ocean [12]. With these issues in mind, the diagram in Figure 2 displays a possible system for generating biomaterials, such as PHAs from waste plastics via fermentation.

Elements of this kind of system exist in parts, such as the optical scanning and separation and sorting of plastics; the innovation would be in having all of these sections interlinked. In some cases, processes such as milling, agglutination, or sonication would have to be selected on the basis of the target material’s properties. After thermal pre-treatments of PS or PE, where oxygenated groups were incorporated into the unsaturated carbon backbone, sonication was found to greatly increase the mixing of plastics with the growth media for fermentation [2,3,4]. Due to the nature of microbial cultivation, requiring time (normally 48hrs or more) and optimal growth conditions (usually ranging from 30 to 37 °C at 50 to 150 rpm), a lot of energy is needed [4,10]. This is perhaps the major bottleneck for such a system, which is why the choice of micro-organisms used is so important. Additionally, any biological PHA generation has the often-unreported issue of difficulty in controlling the precise purity and biopolymer composition. Moreover, there is evidence showing that the extraction processes can alter the PHA-polymer structure when conventional chemicals (such as chloroform) and Soxhlet extraction are used [13]. The extraction yields and the PHA properties can also be affected. Data revealed the two different extraction methods alter the crystallization degree and the chemical composition. When pure bioplastic is required, pre-treatments such as homogenization provided a 15% more extractive yield than the others, especially at high pressures, which also improved the visual appearance (transparency and clearness), thermal stability, and mechanical performances, which is ideal for medical grade PHAs [13]. For packaging (the major application of PHAs), these polymers have already been proposed to effectuate a significant shift in the industry, which currently utilizes almost 40% of plastics created [14].

## 2. Microbes of Interest

Several microbes and isolated enzymes have been reported to have the ability to break down petrol-based plastics. Here we will focus on PP, PS, and PE degradation by micro-organisms, as well as some of the microbial metabolic pathways of the plastic depolymerization products. As mentioned, a number of bacterial strains can degrade polyolefins (simple alkenes), and they are often wild-types found in soil, marine water, sewage sludge, landfills, and even the guts of plastic-eating worms [3,8]. Table 1 contains a list of bacteria and the polyolefin substrates they can utilize.

It is worth noting some fungal species that have been discovered, such as *Engydontium album*; however, with an incubation time of a year on PP, any practical use in a competitive industrial setting would be limited [30]. Other fungi examples with shorter incubation periods of up to 28 days include *Zalerion maritimum*, although this was with PE micro-particles [31]. The future of microbial research in this area is likely to progress further in the direction of the genetic engineering of native species. A marine bacterium was transformed into a hydrocarbon degrader through the transfer of genes from the hydrocarbon degrader *P. putida* [32]. Similar, carefully monitored, genetic engineering approaches (such as Zinc finger proteins, TALENs, and the CRISPR/Cas9) could be applied to unify the functions of genes and enzymes that allow plastic degradation and bioremediation [32].

## 3. Target Plastics

### 3.1. Polypropylene

PP is the second-most widely manufactured petrol-based plastic after PE, and some of its uses include sutures, banknotes, bottles, and packaging [5,33,34]. PP is cheap and strong, yet lightweight; however, its stability makes natural biological degradation difficult. As a result of this, PP production creates a large carbon footprint, and, because of the very short lifespan of packaging materials, most PP finds its way into landfill sites [32,33]. The microbial breakdown of PP was first assessed in 1993 using strains originating from sandy soils that contained waste PE with an incubation period of 175 days [9]. Approximately 90% of the extracted product was found to be aromatic esters (derived from the plasticizers), while 10% was classified as hydrocarbons (C10 to C31) that could be from the PP [33]. Since that time, other bacterial strains have been found, and they feature in Table 1. There has been a recent trend within the scientific community to move towards the incorporation of “mixed consortia” to aid with the breakdown of difficult and highly stable waste material. Four bacterial isolates, from various waste landfills and sewage treatment plants, were used over 140 days to biodegrade PP with a weight loss of 44 to 56% [15,35]. The microbe *Stenotrophomonas panacihumi* PA3-2 was discovered to have the ability to degrade PP samples of lower molecular weights, 2800 and 3600 Da, and of higher mass, 44,000 Da [29,35]. So far, despite some reports of PP breakdown, there are some doubters that PP enzymes exist, or at least believers that the evidence provided is not convincing, and there is not much understanding of the mechanisms involved [35,36,37]. It is certainly likely that some reports of untreated PP degradation by enzymes and microbes were partially deceived by the breakdown of chemical additives, rather than the polymer itself [33,38,39]. Alternatively, physicochemical pretreatments such as UV or γ-irradiation and thermo-oxidation can enable PP degradation with bacteria [4,30,35,40]. It has been shown that applying thermal treatments can allow bacteria, such as *Cupriavidus necator*, not only to breakdown the oxidized (and therefore more hydrophilic) PP samples in nitrogen rich environments, but also to provide a carbon source for PHA synthesis; specifically, PHBH (copolymers of 3-hydroxybutyrate and 3-hydroxyhexanoate) as demonstrated by ES-MS/MS [4,41].

### 3.2. Polystyrene

**Figure 3:**
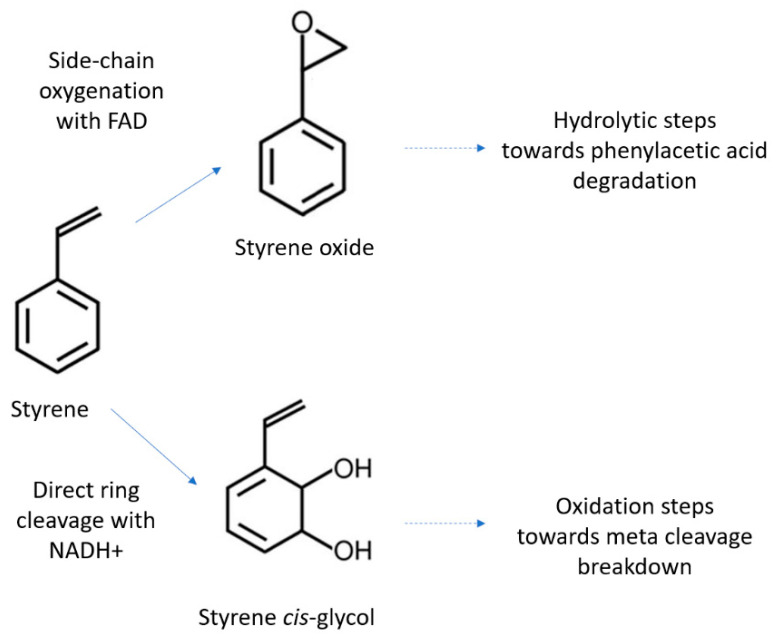
Two major aerobic degradation routes of styrene in microorganisms such as *Pseudomonas*. Essential coenzymes FAD = Flavin Adenine Dinucleotide (an essential coenzyme) and NAD = Nicotinamide adenine dinucleotide, required for metabolism.

PS poly(1-phenylethene) polymers consist of styrene monomers, and this thermoplastic polymer is largely used for packaging, as well as disposable items such as Petri dishes [3]. A large number of bacterial genera are known to be able to metabolize the styrene monomer [33]. In fact, the metabolism of styrene is well understood in bacteria, especially in *Corynebacterium*, *Pseudomonas*, *Xanthobacter*, and *Rhodococcus* [26,28,32,33,42,43]. When bacteria are in aerobic environments, styrene has been found to be oxidized by two pathways: the attacking of the vinyl side-chain and an unspecific aromatic ring. This creates the intermediates 3-vinylcatechol, phenylacetic acid, and 2-phenylethanol, which feed into the Krebs cycle once ring cleavage is performed [33,42,43] (Figure 3).

The degradation of the vinyl side-chain uses three main enzymes: a styrene monooxygenase, a styrene oxide isomerase, and a phenylacetaldehyde dehydrogenase [44]. The styrene monooxygenase cleaves the vinyl side-chain, releasing epoxystyrene, which is then converted into phenylacetaldehyde and eventually phenylacetic acid via oxidation [44]. The metabolism of PS in other bacterial species, such as *P. putida*, follows the activation of phenylacetic acid to phenylacetyl-CoA and then *ß*-oxidation in order to produce acetyl-CoA. The CoA then feeds into the Krebs-cycle in a similar fashion to the breakdown of thermally oxidized, pre-treated PS fragments [3,33,35].

It has also been reported that brown-rot fungi are capable of breaking down polystyrene, using hydroquinone-driven Fenton reactions [33,35]. Both *Gloeophyllum striatum* DSM 9592 and *Gloeophyllum trabeum* DSM 1398 demonstrated clear depolymerization of PS and relatively quickly, after 20 days under controlled conditions: with the most active *Gloeophyllum* producing almost a 50% reduction in the molecular weight of samples [33,35].

### 3.3. Polyethylene

**Figure 4:**
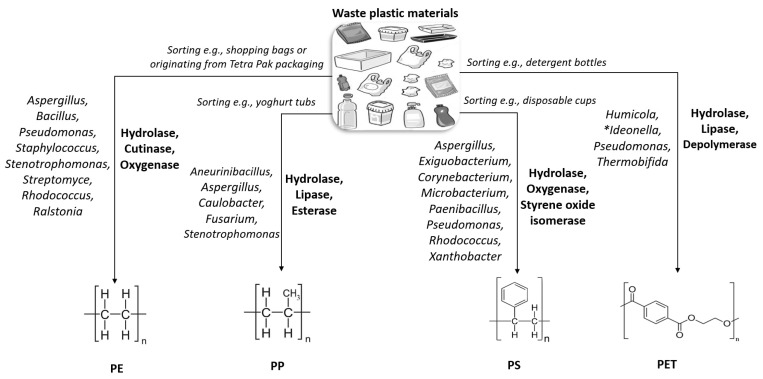
Microbes and major enzymes (in bold) involved in plastic material breakdown. The biochemical pathways involved with *Ideonella are explained further in Section 3.5.

PE consists of long-chain polymers of ethylene, and it is commonly found as either high-density (HDPE) or low-density polyethylene (LDPE). PE usually has a semi-crystalline structure, making it very resistant to biodegradation. Most LDPEs are often utilized for shopping bags, frozen food bags, bubble wraps, and squeezable bottles. The chemical structure is similar to that of HDPE, except that there is more branching. The degradation of PE has been linked with a wide variety of bacterial strains, some of these microbes include Gram-negative *Pseudomonas*, *Ralstonia*, and *Stenotrophomonas*; as well as Gram-positive *Rhodococcus*, *Staphylococcus*, *Streptomyce*, and *Bacillus* [44,45]. In the majority of the studies cited, regarding PE-degrading microbes, the authors reported polymer degradation with commercial polymers that may have contained additives, and the degradation was measured by FTIR readings and weight loss [2,15,25,27,45]. As this change in mass is probably due to the decay of chemical additives (which could compose a large proportion of the polymer structure), the data from these articles could be misleading without further investigation. PE degradation features in Figure 4, where other effective enzymes and microbes are included [22,23,26,27,28,29,32,33,42,43].

### 3.4. Tetra Pak

Tetra Pak^®^ (TP) is a multi-layered, aseptic packaging material that allows liquid food to retain its color, natural taste, nutritional value, and texture for several months without the need for preservatives or refrigeration [46]. TP is composed of three raw materials; cardboard paper (75%), low-density polyethylene (LDPE-20%), and aluminum (Al-5%), structured in six layers [47]. Due to its short life-cycle, the amount of Tetra Pak waste generated is continually on the increase, resulting in serious environmental problems. Thus, the conversion of TP waste into valuable products is simultaneously important for the economy and environment. Ironically, all three components of TP are recyclable, but as a composite material, the recycling of these components is very difficult. Current recycling processes yield a paper fiber and a compound mixture of Al and PE [47]. While there have been no reports on the microbial digestion of whole TP, there have been few reports on the microbial digestion of its individual components. All three component materials are highly recalcitrant and require a synergistic relationship between specialized microbes and secreted enzymes to effectively break them down into simpler substrates [48]. Waste cardboard paper could be utilized for anaerobic digestion, usually by a consortium of mixed microbes and their enzymes, to produce biogas, which could be further upgraded to make chemicals, fuels, and electricity [48]. Anaerobic digesters secrete cellulases consisting of β -glucosidases, endoglucanases, and exoglucanases to efficiently degrade cellulose—the linear polymer present in waste cardboard papers [48]. Prominent cellulose degraders include several bacteria genera such as *Clostridium*, *Fibrobacter*, *Lactobacillus*, *Proeobacteria*, and *Enterococus* [48,49]. Few yeast cells, with *Saccharomyces cerevisiae* being a representative, have also been discovered to anaerobically digest cardboard paper in two pathways [50]. The first pathway involved the use of yeast cells as a source of nitrogen (as waste yeasts are rich in this nutrient) to facilitate digestion and in the second pathway, where *Saccharomyces cerevisiae* fermented polysaccharides into smaller molecules to accelerate digestion process [50]. Ferreira et al. [51] also reported that yeast could establish an effective co-metabolic system with bacteria by providing a sufficient nitrogen source for the anaerobic digestion of cardboard papers.

Concerning the removal of aluminum, the bioleaching process and the use of soil microbes and microbial inoculum can be employed. This microbial process has been found helpful in metabolizing aluminum into less environmentally toxic compounds [52,53]. Some of these microbes include *Bacillus*, *Burkholderia*, and *Thiobacillus ferroxidans* [52,53]. As earlier mentioned in Section 3.3, the PE component of TP can also be potentially degraded by a broad range of microbes. In a recent study by Ekere et al. [54], it was confirmed that *Cupriavidus necator* has the ability to utilize the PE component in TP waste packaging materials for the synthesis of PHA copolymers (3-hydroxybutyrate, 3-hydroxyvalerate, and 3-hydroxyhexanoate) by using accelerator mass spectrometry (AMS). The characterization of the PHAs produced were confirmed to contain 96.73% modern carb on and 3.27% old carbon (derived from Tetra Pak^®^). Thus, the investigation demonstrated the feasibility of using 14C analysis to validate a bioconversion process. However, a solvent-method separation technique was employed in this study to separate the PE component from the waste TP packaging material [54]. The *Pseudomonas* species possess the unique ability of being able to degrade and metabolize PE with extracellular oxidative and/or hydrolytic enzymatic activity that eventually facilitates the uptake and degradation of PE fragments [55]. The precise biochemical pathways and enzymes involved in PE degradation are still quite unknown, and, in most cases, an initial pretreatment step is required to facilitate degradation [55,56].

### 3.5. Poly(ethylene Terephthalate) 

Poly(ethylene terephthalate) (PET) is another major synthetic plastic, and it is produced worldwide in large amounts. By using bio-upcycling routes, PET hydrolysis can be catalyzed by thermostable polyester hydrolases, and those monomers can be fed to an engineered *P. putida*, which can synthesize either extracellular hydroxyalkanoyloxy-alkanoates (HAAs) or PHAs. From the particles of all these waste plastics mentioned, the olefins (via fermentation in appropriate nutrient conditions) can generate PHAs to varying degrees of success.

**Figure 5:**
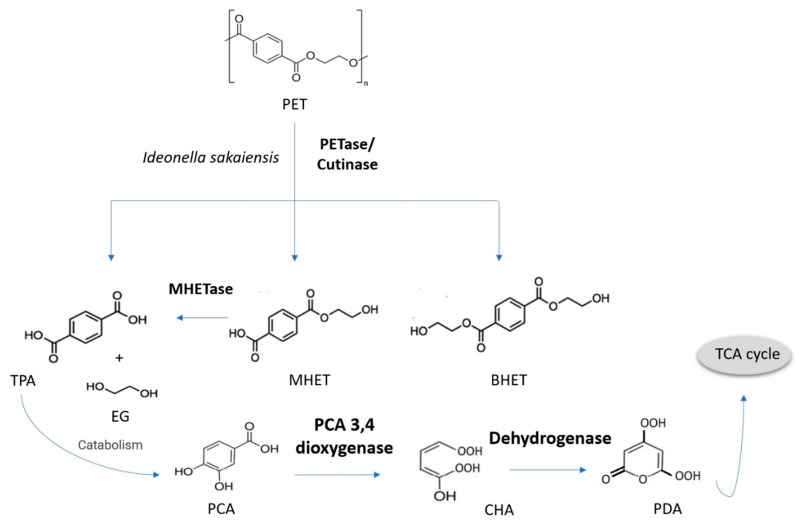
The microbial degradation of PET with key enzymes highlighted in bold. MHET = degrade mono-(2-hydroxyethyl) terephthalate, BHET = bis (2-hydroxyethyl) terephthalate, TPA = to terephthalic acid, EG = ethylene glycol, PCA = protocatechuic acid, CHA = 4-carboxy-2-hydroxymuconic acid, PDA = 2-pyrone-4,- 6-dicarboxylic acid and TCA = tricarboxylic acid cycle.

Some of the microbes mentioned previously that can produce hydrolases could be applied here; PET hydrolase produced from the *I. sakaiensis* genome has been found to code for another novel enzyme that is similar to tannases and is therefore able to degrade mono- (2-hydroxyethyl) terephthalate or MHET as featured in Figure 5 [32,57].

By working in conjunction, these two enzymes can further degrade PET to terephthalic acid (TPA), MHET, and bis (2-hydroxyethyl) terephthalate (BHET). As shown in Figure 5, MHET is further hydrolyzed by MHETase to TPA and ethylene glycol (EG). TPA can then be metabolized to protocatechuic acid (PCA), and then 2-pyrone-4,6-dicarboxylic acid before entering the TCA cycle, where it could be converted into pyruvate, oxaloacetate, and eventually carbon dioxide and water. An industrial application of this process could make plastic solid waste (PSW) a carbon source to produce value-added bioplastic material. However, the degradation of high-molecular-weight and highly robust polymers such as PET and their crystalline structure will remain a challenge for some time [32,57].

## 4. Value-Added Bioplastic Synthesis

The issues of many of the current ways of managing waste plastics (burning and landfill) can often lead to the creation of secondary pollution events. The techniques featured in Figure 2 employ large amounts of energy, which are generally not environmentally friendly or financially viable. Due to poor recycling strategies globally and the durable properties of plastic materials, serious environmental issues, such as oceanic and soil pollution are happening [20]. For these reasons certain plastics have gained attention as carbon sources, especially those that need milder temperatures and less energy consumption for their pre-treatments.

PHAs have attracted a lot of attention mainly due to their similarities to petrochemical polymers, such as those mentioned previously, which makes them a sustainable alternative for a wide range of uses. They can be dissolved in chlorinated solvents and PHAs show a range of properties, from brittle thermoplastics to gummy elastomers, depending on the nature of the fermentation conditions and the carbon-source metabolized by the PHA producer organism [58,59,60]. The structure and the composition of the biopolymers dictate the degradation rate in the environment, and the microbes that generate PHAs cover a broad range, including both Gram positive and Gram negative bacterial strains, as shown in Table 2.

**Figure 6:**
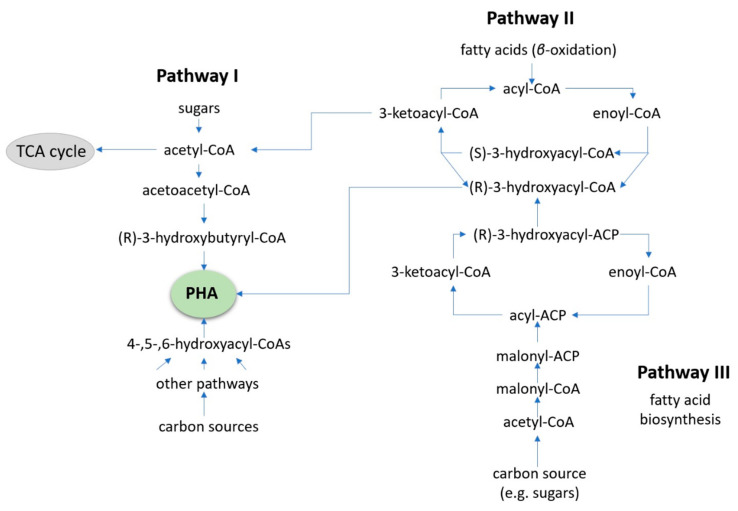
The combined pathways I, II, and III used by *C. necator* for PHA bioconversion. Similar biochemical pathways would be expected for other bacterial species capable of PHA synthesis where oxidized PS, PE, or PP waste plastic is treated as a fatty acid.

Gram-negative *Cupriavidus necator* has been shown to have an accumulation yield of up to 90% cell dry weight, and, for this reason, it is the most studied microbe for PHA production [71]. The metabolic pathways used by *C. necator* are well-documented, both for aerobic and anerobic conditions. In the cases of alternative carbon substrates (such as pretreated plastics), biochemical pathways I, II, and III can be selected by the organism. Figure 6 displays these pathways and how they intersect. These pathways are similar to those utilized by *Bacillus megaterium*, which can accumulate up to 62% cell dry weight, a different archaea species from the family *Halobacteriaceae* that has also been found to produce PHA biopolymers [72,73,74,75,76].

Glucose and fructose are normally processed in pathway I, generating PHB homopolymers. Fatty acids or sugars are metabolized via pathways II, III, or potentially other routes where copolymers can be produced [77,78,79]. Oxidized PE, PS, and PP particles are thought to enter the *β*-oxidation pathway forming acetyl-CoA that is then metabolized along pathway I, creating PHA polymers [75,76]. Both pure and mixed cultures (such as a cascade set-up with biosurfactant or hydrolase synthesizers) have the ability to make use of waste materials as feedstock to produce value-added PHAs. This approach, combined with the use of and using locally sourced refuse, could contribute to vastly reducing bioplastic expenses. PHAs are a good alternative to traditional plastics, but they have a long way to go before they can surpass them, due to their high production costs, lack of specific policies, and the downstream processing [76,79]. With that said, the next evolution in bioconversion to produce PHAs is likely to be focus towards extremophile strains. The reason for this is that they combine pure culture advantages (easier optimization of conditions), plus time can be saved by working in non-sterile conditions, simplifying the extraction and reducing the running costs.

## 5. Conclusions

The bioconversion of plastics (petroleum-derived polymers) is a complicated process with multiple variables. The detection of micro- and nano-plastics in our waters means the pollution issue is much more personal than ever before. Due to biodegradation, thermo-oxidative degradation, photodegradation, thermal, and hydrolysis processes in the ecosystem, there is a major threat to sea-life and humans indirectly. The carbon backbone of plastics means they are very hydrophobic and inert in nature; however, by utilizing physical and chemical methods, micro-organisms have demonstrated various routes for degradation. Most importantly, non-conventional feedstocks are being applied and researched globally, and this will contribute to the development of circular economy systems [10]. In this article, several bacterial strains were highlighted that are capable of breaking down a variety of plastics, namely PS, PP, and PE, and some of the pretreatment techniques that could be applied.

In some cases, commercially available polymers and films could be used as substrates (pre-treated or not). They may be composed of certain plasticizers, additives, or biodegradable impurities, which are more facile for microbial use than the actual carbon backbone itself, as waste fungal biomass from other processes could potentially act as a carbon source for bacterial growth for the purpose of plastic breakdown. This could lead to false positives being reported in some of the studies referenced. Therefore, the analysis of plastics undergoing fungal, enzymatic, or bacterial breakdowns requires further standardization.

Further use of synthetic biology and mixed microbial cultures, in conjunction with metabolic engineering tools to generate microbes, will develop these *value-added products* for the post-consumer and contribute to a further improved model of production and consumption. Unfortunately, the slower functionality of biological systems is currently limiting this process. Long-term, coordinated clean-up operations are required to evaluate the detrimental ecosystem effects of plastics, and the use of locally sourced waste for bioconversion could be another way carbon footprints are reduced, i.e., by allowing the monomers and oligomers formed after breakdown to be utilized to create more suitable, biodegradable, and diverse commodities, particularly in developing countries.

## Figures and Tables

**Table 1:** Bacterial strains and polyolefins metabolized.

Strain	Isolated Source	Plastic Substrate	Days Incubation	References
*Achromobacter xylosoxidans*	Soil	HDPE	150	[15]
*Alcanivorax borkumensis*	Mediterranean Sea	LDPE	7	[16]
*Aneurinibacillus aneurinilyticus*	Landfill or sewage sites	PP	140	[17]
*Bacillus* sp. strain 27	Mangrove environments	PP microplastics	40	[18]
*Bacillus* sp. YP1	Waxworm guts	LDPE film	60	[19]
*Bacillus subtilis* H1584	Marine water	LDPE film	30	[20]
*Brevibacilus argi*; *Brevibacilus brevis*; *Brevibacilus* sp.	Sewage	PP	140	[17]
*Cupriavidus necator*	Soil	LDPE, PP, PS	2	[3,4,21]
*Exiguobacterium* sp. YT2	Mealworm guts	PS film	60	[22]
*Microbacterium* sp. NA23	Soil	PS film	56	[23]
*Paenibacillus urinalis* NA26	Soil	PS film	56	[23]
*Pseudomonas* sp. AKS2	Soil	LDPE	45	[24]
*Pseudomonas* sp. E4	Soil	LMWPE	80	[25]
*Rhodococcus ruber* C208	Soil	PS film	56	[26]
*Rhodococcus* sp. strain 36	Mangrove environments	PP	40	[18]
*Serratia marcescens*	Soil	LLDPE film	70	[27]
*Sphingobacterium* sp.	Field soil	PS film	8	[28]
*Stenotrophomonas panacihumi*	Soil	PP film	90	[29]
*Xanthomonas* sp.	Field soil	PS film	8	[28]

LMWPE = Low molecular weight polyethylene; LLDPE = Linear low-density polyethylene.

**Table 2:** Notable research of bioplastic production from the last 15 years on Gram-positive (+) and Gram-negative (−) bacterial strains, with their respective carbon sources and biopolymers produced. * Bio-PU a novel bio-based poly (amide urethane).

Strain	Carbon Source	Polymer Synthesised	References
*Bacillus megaterium* (+)	Glucose salt medium	PHB	[61]
*Bacillus* spp. (+)	Soy molasses, nutrient broth, glucose, butyrate, valerate, hexanoate, octanoate, decanoate, 4-hydroxybutanoate, e-caprolactone	PHB, PHBV, copolymers	[61]
*Burkholderia cepacia* (−)	Palm olein, palm stearin, crude palm oil, palm kernel oil, oleic acid, xylose, levulinic acid, sugarbeet molasses, sugar maple hemicellulosic hydrolysate	PHB, PHBV	[62]
*Caryophanon latum* (+)	Nutrient broth	PHA	[63]
*Cupriavidus necator* (−)	Glucose, soybean oil, waste PE, PP, PS, plastics, biodiesel by-product substrates	PHB, PHBV, PHBH, PHBHx, copolymers	[2,3,4,21,54,56,64,65]
*Caldimonas taiwanensis* (−)	Potatoe and wheat starch	PHBV	[66]
*Bacillus odysseyi SUK3* (+)	PS plastic	PHB	[67]
*Haloferax mediterranei* (−)	Molasses and wastewater	PHBV	[68]
*Pseudomonas umsongensis* GO16 (−)	Ethylene glycol	PHA, * Bio-PU	[69]
*Zoogloea* spp. (−)	Nutrient broth (activated sludge/wastewater)	PHA	[70]

## Data Availability

Not applicable.
